# Relationship between dietary intake and erythrocyte PUFA in adolescents from a Western Australian cohort

**DOI:** 10.1038/s41430-022-01219-x

**Published:** 2022-10-07

**Authors:** Fuzhen Wan, Feng Pan, Trevor A. Mori, Therese A. O’Sullivan, Lawrence J. Beilin, Wendy H. Oddy

**Affiliations:** 1 Menzies Institute for Medical Research, University of Tasmania, Hobart, TAS Australia; 2 Medical School, Royal Perth Hospital Unit, University of Western Australia, Perth, WA Australia; 3 Institute for Nutrition Research, School of Medical and Health Science, Edith Cowan University, Joondalup, WA Australia

**Keywords:** Epidemiology, Fatty acids, Membrane lipids

## Abstract

**Background:**

Population-based studies show that the intake of omega-3 (n-3) and omega-6 (n-6) polyunsaturated fatty acids (PUFA) are associated with a range of health conditions. Therefore, the reliability of food frequency questionnaires (FFQ) as rapid and easily accessible screening tools for PUFA intake deserve investigation.

**Objective:**

We aimed to assess the relationship between erythrocyte fatty acids and fatty acid intake collected using the Commonwealth Scientific and Industrial Research Organisation (CSIRO) food frequency questionnaire in an adolescent cohort.

**Design:**

A cross-sectional study using data from 1155 young adolescents participating in the 14-year follow-up of the Raine Study. Bland–Altman plots were used to determine the agreement between dietary intake and erythrocyte levels of each fatty acid.

**Results:**

The main dietary source of n-3 long-chain (LC) PUFA was ‘fresh fish’ (53% of total n-3 LC-PUFA). Docosahexaenoic acid (DHA) showed the strongest correlation between erythrocyte and diet assessment (*r* = 0.274; *p* < 0.001), whilst linoleic acid (LA) (*r* = 0.103; *p* < 0.001) and arachidonic acid (AA) (*r* = −0.06; *p* = 0.042) showed weaker correlations, with limits of agreement relatively narrow. Bland–Altman plots showed a dose-dependent bias between the FFQ fatty acid data and corresponding erythrocyte data.

**Conclusions:**

For the major n-3 and n-6 PUFA, dietary intakes derived from the FFQ showed weaker correlations and poorer agreement with erythrocyte levels, and the deviation between the two increased with higher intake levels.

## Introduction

Dietary polyunsaturated fatty acids (PUFA) are essential for human health and play an important role in many biochemical activities, including but not limited to cell membrane fluidity, signal transition and energy provision within the body [1, 2]. PUFA can be classified as n-3 or n-6 according to the position of the first double bond, i.e. three carbons away from the terminal methyl group or six carbons away from the terminal methyl group. Mammalian cells can synthesise most of the fats required from dietary intake. However, the term ‘essential’ is applied to two PUFA, n-3 alpha-linolenic acid (ALA, 18:3n-3) and n-6 linoleic acid (LA, 18:2n-6), which must be consumed from the diet as the body cannot make these from other fatty acid precursors. Long-chain omega-3 PUFA, such as eicosapentaenoic acid (EPA, 20:5n-3) and docosahexaenoic acid (DHA, 22:6n-3), can be synthesised from ALA; long-chain omega-6 PUFA, such as arachidonic acid (AA, 20:4n-6), can be synthesised from LA. Both ALA and LA play different roles in physiological systems and pathological processes. Modern Western diets typically have a high n-6: n-3 PUFA ratio of 15–16:1, with some research suggesting the ratio may have health implications [3, 4].

Assessing the intake of PUFA in different populations and validating it with biomarkers is important in order to understand the relationship between fat intake and health outcomes. The biological status of PUFA fatty acids can be measured in several sample types including red blood cells, whole blood plasma, platelets, white blood cells and plasma lipid classes. PUFA have different metabolic kinetics in different samples, with free PUFA metabolised in plasma as quickly as a few hours but retained in erythrocytes for weeks to months. Therefore, erythrocytes may be the biomarker of choice for long-term PUFA bio-status in clinical practice and research. Currently, validation studies based on erythrocyte fatty acids and dietary intake from FFQ are inadequate with most coming from adult populations using small clinical trials [5–7]. A limited number of studies have assessed and validated dietary intake of PUFA in adolescents [8–11], with most studies having small sample sizes (*n* = 70–400). Erythrocytes turn over every few months [12, 13] and do not have enzymes for fatty acid metabolism [14]. To our knowledge, erythrocyte fatty acid composition has not been used to validate fatty acid intakes from the Commonwealth Scientific and Industrial Research Organisation (CSIRO) food frequency questionnaire (FFQ) [15] in a young adolescent population. This FFQ was previously validated against other food intake measurement methods such as weighed food records [16–19] and is a tool that has been widely used in Australia.

Our study aimed to use 14 years of follow-up data from the Raine Study to (1) assess dietary intake and food sources of n-3 and n-6 PUFA in a Western Australian adolescent population and (2) measure the relationship between dietary intake of PUFA as determined by the CSIRO FFQ and erythrocyte fatty acid composition.

## Methods

### Population

This study is a cross-sectional analysis of data collected during the 14-year follow up of the Raine Study (2003–2006). The Raine Study is a prospective pregnancy cohort, with 2900 pregnant women attending the public antenatal clinic at King Edward Memorial Hospital or nearby private practices between 16 and 20 weeks gestation. The women were recruited between May 1989 and November 1991 (Gen1), and a total of 2868 babies (Gen2) were eligible for follow up from birth [20]. Follow up assessments of the offspring (Gen2) cohort have been conducted approximately every 3 years, with all participants providing informed written consent. Ethics approval was granted from the Human Research Ethics Committees at King Edward Memorial Hospital, Princess Margaret Hospital for Children and the University of Western Australia, in Perth, Western Australia.

### Dietary intake data

Designed to provide respondents with an analysis of their daily dietary intake, the CSIRO FFQ collects information on the frequency of consumption of 212 food items, mixed dishes and beverages [21]. The FFQ was internally validated for application to the target population by applying a less biased instrument (3-day food records as the ‘gold standard’) and calibrating the FFQ data against it [22]. The FFQ assessed daily dietary intake over the previous 12 months. It consists of two types of questions: a food list where respondents record how often they consume each food item and a question on the amount of servings they usually consume. It was modified to include the favourite drinks and snacks of adolescents and to exclude alcohol. The frequency of consumption options included: never, rarely, number of times per month, number of times per week and number of times per day.

Given the age of the respondent (14 years ± 11 months) and the potential difficulty or lack of interest in completing the FFQ [23], we asked the primary caregiver to complete the FFQ in consultation with the adolescent. Details were requested on cooking methods, types of oil used and whether food was low-fat, fresh, frozen or canned. Respondents were also asked to record any other foods that were regularly consumed but not included in the FFQ. Separate questions were asked about the frequency of specific fruit and vegetable intake during summer and winter. The frequency of food consumption in summer and winter was used to measure seasonal differences. In addition to this, as fish is the major source of long-chain (LC) PUFA (EPA Docosapentaenoic acid (DPA, 22:5n-3) and DHA) in the diet, respondents were asked to name the type of fish they ate most often, allowing for a more detailed measurement of fish n-3 intake. All FFQs were checked by a research nurse, and questions were clarified with the adolescents. Data from the FFQs were analysed by CSIRO (Australia) using Australian food composition data (Food Standards Australia New Zealand, 1995).

### Erythrocyte biomarker data

Blood samples were taken by a trained phlebotomist after an overnight fast. Erythrocytes were isolated from whole blood, washed with isotonic saline (0.9%), frozen, thawed then lysed in hypotonic 0.01-m Tris EDTA buffer, pH 7.4. A membrane pellet was obtained by ultracentrifugation (50,000 × *g* for 30 min) from which lipids were extracted and trans methylated according to the method of Lepage and Roy [24]. Fatty acids were measured by gas chromatography as previously described [25]. Briefly, erythrocyte lipids were extracted and analysed using an Agilent Technologies model 7890A gas chromatograph (Santa Clara, CA). The column was a Supelco SP-2560 (100 m × 0.25 mm ID × 0.20 mm; Bellefonte, PA) with a temperature programme as follows: 180 °C (1.75 min), then 5 °C/min to 200 °C (held 1.75 min), then 10 °C/min to 240 °C (held 4.5 min) using hydrogen as carrier gas at a split ratio of 30:1. Peaks were identified by comparison with a known standard mixture.

### Additional participant data

Questionnaires completed by the participants determined lifestyle and sociodemographic factors. A trained research assistant weighed and measured participants in light clothing for height and weight using a calibrated stadiometer and electronic scales as previously described [26]. Body mass index was calculated as weight (Kg) /height (m^2^). The primary caregivers of the participants were asked to report their annual family income (in Australian dollars) at the 14-year follow-up (2003–2006) and were categorised as: <$30,000, $30,001–50,000, $50,001–78,000, and >$78,000. The proportion with Caucasian mother data were collected at study recruitment 16–20 weeks gestation.

### Statistical analysis

Statistical analysis was conducted using STATA version 16 for Windows (Stata Statistical Software: College Station, Tx, USA). A *p* value of <0.05 was determined to be significant for the results. Means, standard deviations (SD) or percentages were calculated for demographic data and characteristics of the study population.

We used the Shapiro–Wilk test as the normality test in this study. The FFQ and erythrocyte data were assessed for normality and, as most of the data were not normally distributed, non-parametric tests were used. The raw PUFA intake was adjusted for energy using the energy density method (i.e. using PUFA intake by dividing each individual’s total energy intake and multiplying by 9.4 MJ/day (approximately the median total energy intake in cohort) and energy-adjusted data is still not normal after this adjustment). Dietary contributions to n-3, n-6 and n-3 LC-PUFA were calculated by summing the contributions of certain specific food and beverage groupings, of which there were 20 food groups in total and expressed as median daily intake and a percentage of total intake for each PUFA category (calculated at the individual level). Spearman’s rank correlation coefficients (*r*) were used to compare the correlation between energy-adjusted FFQ fatty acid and erythrocyte fatty acid. Energy-adjusted FFQ PUFA was categorised into quintiles and then cross-tabulated with the quintiles of the respective PUFA erythrocyte proportions. Quantile cross-validation was used to transform the dataset into hierarchically categorised data. Inconsistency and agreement in quintile rankings were assessed by calculating the percentage of participants classified as being in the same quintile, the same or adjacent quintile and the opposite quintile (The definition of the opposite quantile is defined as a situation where the grouping is in the highest quartile of one method and the lowest quartile of another method). In addition, Cohen’s weighted kappa statistic (*κ*) and 95% CI were calculated for quintiles of energy-adjusted FFQ PUFA intake and erythrocyte PUFA proportions as they accounted for consistency by chance. Correlations (*r*) and concordance (*κ*) were rated as poor (<0.2), fair (0.2–0.59) or moderate (0.6–0.7) [27].

Agreement between the two methods was assessed using the Bland–Altman plot [28] method, which shows the average difference between two measures for each participant, along with ±1.96 SD of the difference. It shows how far apart the measurements between dietary intake and erythrocyte measures are, where a narrower range between these points represents a greater agreement and a wider range represents poorer agreement.

## Results

A total of 2424 adolescents were eligible to participate in the 14-year follow-up (excluding those who had withdrawn, deferred, or deceased). Of 1864 who participated in the follow-up, 70% (*n* = 1302) provided a blood sample, and 88% (*n* = 1632) completed the FFQ. This study includes the 62% (*n* = 1155) of participants who had both complete dietary intake data and erythrocyte biomarker data.

### FFQ and erythrocyte PUFA

Table 1 summarises the anthropometry and macro-nutrient intake characteristics of the 1155 participants of which 52% (*n* = 603) were male. The mean energy intake was 9.69 (SD 3.04) MJ/day. Fat intake (91.8 g/day) contributed 34.8% to total energy intake. FFQ intakes and erythrocyte ratios (as a percentage of total fat) are shown in Table 2, along with data for PUFA subtypes. The majority of PUFA were consumed as n-6 PUFA with LA predominating; the majority of n-3 PUFA intake was in the form of ALA. Daily intake of DHA was 76 mg/day, with 25% of children <37.2 mg/day.

**Table 1:** Characteristics of participants at the 14 year follow-up of the Raine Study.

Mean ± SD or *n* (%)	All (*n* = 1155)	Male (*n* = 603)	Female (*n* = 552)	*p*
Height (cm)	164.5 ± 7.8	166.4 ± 8.7	162.3 ± 6.0	<0.001
Weight (kg)	57.9 ± 12.9	58.6 ± 13.9	57.3 ± 13.9	0.092
BMI (kg/m^2^)	21.3 ± 4.1	21.0 ± 4.1	21.7 ± 4.1	<0.001^a^
Maternal race (%)	88.8	90.1	87.7	0.170^a^
Annual family income	*n* = 1137	*n* = 593	*n* = 544	0.026^a^
<35,000	270	124	146	
35,000–70,000	415	220	195	
>70,000	452	249	203	
Dietary variables				
Energy (MJ/day)	9.69 ± 3.04	10.5 ± 3.04	8.81 ± 2.83	<0.001^a^
Total fat (g/day)	91.8 ± 33.7	99.7 ± 34.0	83.1 ± 31.2	<0.001^a^
Total fat (% energy)	34.8 ± 5.1	34.9 ± 5.2	34.7 ± 5.1	0.280
Carbohydrate (g/day)	277 ± 90.7	299 ± 90.1	254 ± 85.4	<0.001^a^
Carbohydrate (% energy)	45.9 ± 5.1	45.7 ± 5.0	46.1 ± 5.2	0.150
Protein (g/day)	96.4 ± 30.7	105 ± 30.3	87.4 ± 28.5	<0.001^a^
Protein (% energy)	17.1 ± 2.6	17.1 ± 2.5	17.0 ± 2.7	0.269

A limit of three significant figures for all diet data. *p* values for *t*-tests or Mann–Whitney test. Proportion with Caucasian mother data were collected at study recruitment 16–20 weeks gestation.

*BMI* body mass index.

^a^Means tested by Mann–Whitney test.

**Table 2:** Mean (SD) and IQR values for intake per day and percentage of total fat and erythrocyte fatty acid (*n* = 1155).

Fatty acids (total and subtypes)	Mean	SD	Median	IQR
FFQ data				
n-3 PUFA, g/day	1.29	0.63	1.16	0.88–1.56
ALA, g/day	1.04	0.58	0.89	0.67–1.23
EPA, mg/day	70.9	37.6	64.9	44.2–89.7
DPA mg/day	101	52.7	93.2	62.5–128
DHA, mg/day	76.0	57.5	63.3	37.2–98.5
n-6 PUFA, g/day	11.8	6.12	10.3	7.26–15.4
LA g/day	11.5	6.09	10.0	6.97–15.1
AA mg/day	172	81.6	160	114–215
Erythrocyte data, %				
n-3 PUFA	8.52	1.22	8.57	7.86–9.22
ALA	0.62	0.21	0.59	0.53–0.66
EPA	0.69	0.19	0.69	0.58–0.80
DPA	2.39	0.40	2.39	2.17–2.62
DHA	4.20	0.96	4.20	3.64–4.76
n-6 PUFA	33.1	2.57	33.7	32.3–34.7
LA	9.96	1.03	9.92	9.32–10.6
AA	13.3	1.96	13.7	12.8–14.5

All the variables are not normally distributed.

*AA* arachidonic acid, 20:4n-6, *ALA* α-linolenic acid, 18:3n-3, *DHA* docosahexaenoic acid, 22:6n-3, *DPA* docosapentaenoic acid, 22:5n-3, *EPA* eicosapentaenoic acid, 20:5n-3, *IQR* interquartile range, *LA* linoleic acid, 18:2n-6, *n-3 PUFA* Omega 3 polyunsaturated fatty acids, *n-6 PUFA* Omega 6 polyunsaturated fatty acids, *SD* standard deviation.

### Dietary sources of PUFA intake

The average daily intake and the percentage contribution to n-6 and n-3 PUFA and n-3 LC-PUFA intake according to food group are shown in Table 3. The highest intake of n-6 PUFA came from ‘butter, margarine’ and ‘cereals, breads’, which together accounted for 40% of the n-6 PUFA intake. Other important sources were ‘crisps, snack foods, nuts, seeds’ and ‘savoury dishes, soup, stews’, ‘chicken and fish (excluded fresh fish)’. The main dietary source of n-3 LC-PUFA was ‘fresh fish’ (53% of LC-PUFA), with most other food groups providing nil to minimal amounts. N-3 PUFA was mainly derived from ‘butter, margarine’ and ‘savoury dishes, soup, stews’.

**Table 3:** Daily intake and percentage contribution of n-6, n-3 PUFA and n-3 LC-PUFA intakes by food group as estimated from FFQ (*n* = 1155).

Food groups	n-3 PUFA				n-6 PUFA				n-3 LC-PUFA			
	g/day		%		g/day		%		mg/day		%	
	Median	IQR	Mean	sd	Median	IQR	Mean	sd	Median	IQR	Mean	sd
Cereals, breads	0.07	0.05–0.09	6.48	3.78	1.13	0.79–1.54	12.03	7.03	0.00	0.00–0.00	0.16	1.62
Cakes, biscuits, honey, jam	0.02	0.01–0.04	2.26	2.27	0.17	0.08–0.32	2.26	2.43	0.13	0.00–0.29	0.17	2.01
Desserts, ice cream	0.02	0.01–0.04	2.37	2.59	0.05	0.02–0.09	0.73	0.98	0.00	0.00–0.00	0.00	0.02
Dairy, soy products	0.12	0.06–0.20	11.97	8.19	0.22	0.11–0.38	2.91	3.20	0.00	0.00–0.00	0.00	0.00
Beverages, sugar, milo	0.00	0.00–0.00	0.03	0.03	0.00	0.00–0.01	0.08	0.03	0.00	0.00–0.00	0.00	0.00
Eggs	0.01	0.01–0.02	1.34	1.28	0.18	0.07–0.35	2.26	2.31	5.10	1.96–9.32	1.71	3.25
Red meat, pork	0.11	0.06–0.18	11.11	8.08	0.28	0.15–0.46	3.53	3.35	60.6	32.3–99.0	15.27	9.26
Sausages, ham, bacon	0.05	0.03–0.08	5.05	3.75	0.39	0.21–0.67	4.97	4.25	8.40	4.74–13.3	2.55	2.66
Takeaway foods (not hot chips)	0.08	0.04–0.12	7.74	5.66	0.35	0.19–0.55	4.22	3.61	10.2	4.38–17.9	3.20	3.23
Savoury dishes, soup, stews	0.15	0.08–0.23	14.69	11.00	0.54	0.31–0.86	6.73	5.85	63.0	32.6–109	16.50	9.49
Chicken, fish (excl fresh fish	0.05	0.03–0.10	6.11	6.20	0.46	0.22–0.84	6.12	6.29	23.0	9.37–43.3	7.00	6.41
Canned, pickled veg, coleslaw	0.00	0.00–0.01	0.46	1.07	0.10	0.02–0.26	1.80	2.36	0.00	0.00–0.00	0.00	0.00
Cooked vegetables, hot chips	0.04	0.02–0.06	3.62	2.50	0.48	0.29–0.72	5.32	3.50	0.00	0.00–0.00	0.00	0.00
Salad vegetables, veg juice	0.00	0.00–0.00	0.00	0.00	0.00	0.00–0.00	0.50	1.48	0.00	0.00–0.00	0.00	0.00
Fruit and fruit juice	0.00	0.00–0.00	0.00	0.00	0.00	0.00–0.00	0.00	0.00	0.00	0.00–0.00	0.00	0.00
Crisps, snack foods, nuts, seeds	0.00	0.00–0.04	4.48	11.59	0.43	0.14–1.01	7.79	11.20	0.00	0.00–0.00	0.00	0.00
Confectionery, ice cream bars, icy pole	0.02	0.01–0.03	2.11	1.80	0.37	0.16–0.65	4.79	4.93	0.00	0.00–0.00	0.00	0.00
Sauce, dressing, dips, spreads	0.00	0.00–0.01	0.57	1.21	0.35	0.00–0.70	5.06	7.00	0.00	0.00–0.00	0.00	0.00
Butter, margarine	0.17	0.09–0.31	18.16	15.38	3.09	0.28–6.78	28.86	23.11	0.00	0.00–0.00	0.00	0.00
Fresh fish	0.01	0.00–0.02	1.42	1.84	0.00	0.00–0.00	0.02	0.04	227	157–315	53.45	4.28

A limit of three significant figures for all diet data. The n-3 PUFA is made up of ALA, EPA, DPA and DHA data; the n-6 PUFA is made up of LA, AA data; the n-3 LCPUFA is made up of EPA, DPA and DHA data.

*IQR* interquartile range, *n-3 PUFA* Omega 3 polyunsaturated fatty acids, *n-6 PUFA* Omega 6 polyunsaturated fatty acids, *SD* standard deviation, *LC PUFA* long chain omega 3 polyunsaturated fatty acids.

### Validation analysis

The results of Spearman’s correlation are shown in Table 4. Overall, energy-adjusted FFQ intake of LA, DPA and DHA showed a significant correlation with their respective erythrocyte levels. The strongest correlation was between the two measures of DHA (*r* = 0.316, *p* < 0.001). Regarding the correlation between the different types of PUFA, dietary LA was not correlated with erythrocyte AA concentrations. For the n-3 PUFA, there was a weak positive correlation between dietary ALA and erythrocyte EPA. There was also a weak positive correlation between dietary EPA and DHA erythrocyte levels.

**Table 4:** Correlation data at 14 year of age in the Raine Study: nutrient intake (g/day) against erythrocyte measures and percentage fatty acid intake from total fat and percentage of erythrocyte measures using an energy-adjusted rho (*ρ*) correlation (*n* = 1155).

FFQ	Erythrocyte											
	n-6 PUFA				n-3 PUFA							
	LA rho (*ρ*)	*p* value	AA rho (*ρ*)	*p* value	ALA rho (*ρ*)	*p* value	EPA rho (*ρ*)	*p* value	DPA rho (*ρ*)	*p* value	DHA rho (*ρ*)	*p* value
n-3 PUFA	0.076	0.010	−0.037	0.215	−0.035	0.241	0.074	0.011	−0.027	0.364	0.115	0.001
ALA	0.078	0.008	−0.051	0.084	−0.015	0.603	0.074	0.012	−0.035	0.231	0.046	0.120
EPA	−0.034	0.242	−0.038	0.196	−0.026	0.382	0.027	0.363	−0.063	0.031	0.167	<0.001
DPA	−0.020	0.495	−0.006	0.846	−0.022	0.455	−0.030	0.314	−0.074	0.012	0.080	0.006
DHA	−0.024	0.407	−0.057	0.051	−0.041	0.164	0.143	<0.001	−0.079	0.007	0.316	<0.001
n-6 PUFA	0.096	0.001	−0.033	0.266	−0.081	0.006	−0.040	0.173	−0.037	0.206	−0.005	0.867
LA	0.096	0.001	−0.033	0.269	−0.080	0.007	−0.037	0.212	−0.035	0.229	−0.005	0.868
AA	−0.041	0.161	0.006	0.829	−0.024	0.413	−0.059	0.044	−0.107	0.003	0.075	0.011

*AA* arachidonic acid, 20:4n-6, *ALA* α-linolenic acid, 18:3n-3, *DHA* docosahexaenoic acid, 22:6n-3, *DPA* docosapentaenoic acid, 22:5n-3, *EPA* eicosapentaenoic acid, 20:5n-3, *LA* linoleic acid, 18:2n-6, *n-3 PUFA* Omega 3 polyunsaturated fatty acids, *n-6 PUFA* Omega 6 polyunsaturated fatty acids.

Cross-classification analysis of quintiles of FFQ and erythrocyte PUFA subtypes showed that 50–61% of adolescents were classified into the same or adjacent quintiles with the highest concordance for DHA (Table 5). In contrast, 4–9% of participants were misclassified into the opposite quintile. Kappa statistics (Table 5) showed poor agreement (*κ* < 0.2) between their respective dietary and plasma measurements for all n-6 and n-3 PUFA. The highest agreement was found between FFQ and erythrocyte DHA (*κ* = 0.174, *p* < 0.001).

**Table 5:** Energy-adjusted dietary PUFA intakes classified into quintiles compared with quintiles of erythrocyte PUFA proportions with corresponding Cohen’s *κ* coefficients (*n* = 1155).

FFQ and erythrocyte PUFA	Same quintile (%)	Same or adjacent quintile (%)	Opposite quintile (%)	Cohen’s Kappa (*κ*)		
				Cohen’s *κ*	95% CI	*p* value
n-3 PUFA	23.81	50.06	7.36	0.061	0.020–0.102	0.004
ALA	20.35	54.81	8.57	0.004	−0.036–0.044	0.846
EPA	22.34	53.07	7.10	0.022	−0.019–0.062	0.296
DPA	17.40	49.96	7.79	−0.054	−0.092–−0.015	0.007
DHA	27.97	62.51	3.46	0.198	0.158–0.239	<0.001
n-6 PUFA	21.21	52.73	8.14	0.021	−0.019–0.061	0.311
LA	22.08	55.15	6.75	0.063	0.023–0.103	0.002
AA	21.39	50.65	8.40	0.002	−0.038–0.042	0.924

Cohen’s Kappa analysis using the weighted Kappa statistic (*κ*).

*AA* arachidonic acid, *ALA* α-linolenic acid, *DHA* docosahexaenoic acid, *DPA* docosapentaenoic acid, *EPA* eicosapentaenoic acid, *LA* linoleic acid, *n-3 PUFA* Omega 3 polyunsaturated fatty acids, *n-6 PUFA* Omega 6 polyunsaturated fatty acids.

**Fig. 1:**
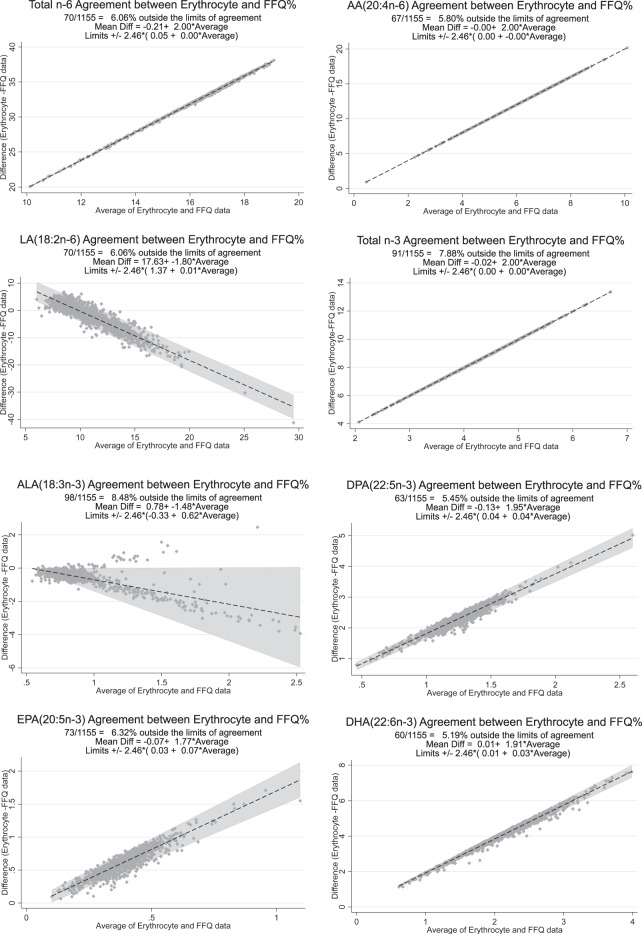
Bland–Altman plot, showing 95% limits of agreement for ALA, LA, EPA and DHA in population. EPA Eicosapentaenoic acid, ALA α-Linolenic acid, DHA Docosahexaenoic acid, LA Linoleic acid, FFQ Food Frequency Questionnaire.

The Bland–Altman plots for each of the fatty acids, comparing the agreement between the dietary fatty acid and the erythrocyte percentages, are shown in Fig. 1. For all fatty acids, the Bland–Altman plot suggests poor agreement between the results determined by the two methods (FFQ% vs. erythrocyte%). Further, the observed slope suggests there was evidence of proportional bias, indicating that the bias between methods did not agree equally through the range of measurements. In all cases, the degree of difference between methods increased as values increased. In addition, erythrocyte DHA% and EPA% levels were consistently higher than these % levels as measured from dietary intake.

### Gender comparisons

We have conducted subgroup analyses by gender and obtained similar results to Table 2. There were significant gender differences in ALA, n-6 PUFA and AA from erythrocyte data (Supplementary Table 1). As with Table 3, the fresh fish food group contributing the majority of the n-3 LC PUFA diet data for both males and females (Supplementary Tables 2 and 3). Correlation analysis between energy-adjusted dietary data and erythrocyte data showed significant gender differences: LA was positively associated in males and DPA negatively associated in females (Supplementary Tables 4 and 5). Male and female subgroups showed similar results in the cross-classification analyses and Bland–Altman plots (Supplementary Tables 6 and 7).

## Discussion

In this study, we examined the weak positive correlation between dietary PUFA from the CSIRO FFQ and their corresponding erythrocyte concentrations. Among all dietary PUFA subgroups, dietary DHA and erythrocyte DHA concentrations had the highest correlation and the highest level of agreement. N-6 PUFA, the PUFA consumed in the largest proportion, resulted in a dietary n-6:n-3 ratio of ~9.1:1. Bland–Altman plots showed that as dietary intake increased and decreased, erythrocyte measurements changed accordingly, although the strength of correlation for each measured fatty acid differed.

In this study, the daily intakes of n-6 and n-3 PUFA and their subtypes (n-3, n-6, LA, AA, ALA, EPA, DPA and DHA) were comparable to those reported in other paediatric populations in Western countries [10, 29–34]. The main food groups contributing to n-3 and n-6 PUFA intakes were very similar between our study and a study of a younger UK school-age population (4–10 years) [29]. In our study, ‘butter, margarine’, ‘cereals and bread’ products contributed the most to n-6 PUFA intake, which is consistent with reports from a 7-year-old UK cohort of 8242 children [29]. The main food source of DHA being fresh fish was also consistent with previous studies [29, 35, 36]. Our study suggests that the intake of DHA in this study population is below the dose recommended by Australian guidelines. According to the recommendations of the Institute of Studies on Fatty Acids and Lipids (ISSFAL), a minimum intake of 200 mg of DHA per day during pregnancy and lactation is recommended [37]. 2021, the National Health and Medical Research Council approved an update to the Australian Pregnancy Care Guidelines, with the recommended measurement of DHA rising to 800 mg per day. This updated recommendation is significantly higher than the 2006 Australian and New Zealand Nutrient Reference Values of 110–115 mg/day for EPA, DHA and DPA. In our study, the ratio of n-6: n-3 PUFA is 9.1:1. This ratio reflects the high proportion of LA food sources in modern Western dietary patterns, including butter, margarine and LA-rich vegetable oils (widely used in processed grain products such as baked and fried foods and snacks). At the same time, fewer food sources with high n-3 PUFA content worsen the ratio of PUFA balance. However, the American Heart Association supports an energy intake of at least 5 to 10% of n-6 PUFA and does not recommend reducing omega-6 PUFA intake on top of this [38]. Therefore, changes in dietary habits to improve PUFA balance by increasing n-3 PUFA intake is necessary, especially for younger age groups, given the long-term cardiovascular and metabolic benefits [39, 40].

Our findings are in accordance with previous reports that have shown PUFA intake from FFQ and their respective biomarkers exhibited poor-to-moderate correlations [29, 41–44]. Our study shows that DHA has the highest correlation between dietary intake and erythrocyte levels, albeit lower than that found in other studies (*r* = 0.34–0.61) [14, 29, 45–47]. A small cohort (female *n* = 33, male *n* = 20) of Australian university staff and students showed a DHA correlation coefficient of *r* = 0.39 between erythrocyte and FFQ data [47]. In Switzerland [46], 152 healthy male and female participants aged between 18 and 59 years showed a strong correlation between the two measures of DHA levels (*r* = 0.605), However, 9% of the participants were recorded as being using supplements. A large cohort study with long-term follow-up showed that the correlation between the two measures of DHA levels fluctuated between *r* = 0.41 and 0.56 across three follow-ups in a US population of female registered nurses between the ages of 30–55 years [14]. In another UK cohort of school-aged children who also used plasma DHA levels as an indicator, the authors found that DHA remained the most strongly correlated of all fatty acids in both dietary and plasma data (*r* = 0.34, *p* < 0.001) [29]. Despite the different FFQs used in these studies above, and even the use of erythrocyte fatty acids in some and plasma fatty acids in others, all showed better correlations for DHA levels compared to other fats. This suggests that FFQ data may be a good tool for measuring DHA levels in the body.

Our data show a weak correlation between FFQ and erythrocyte ALA, EPA and AA. The correlation between fatty acids in human tissues and the intake measured by FFQ is unlikely to be a perfect match, considering that some fatty acids can act as metabolic precursors for other fatty acids. Studies of ALA metabolism in healthy young populations have shown that young women have a greater ability to generate n-3 LC-PUFA from ALA, with ~21% of dietary ALA converted to EPA and 9% to DHA [48], compared to ~8% of dietary ALA converted to EPA and 0–4% to DHA in men [49]. This conversion depends on factors such as gender, pregnancy and diet [50, 51]. For example, vegetarians tend to be more efficient converters than non-vegetarians. In addition, because erythrocytes have a metabolic lifespan of ~120 days, it is thought that erythrocyte membrane fatty acids reflect fatty acid metabolism levels over a longer time span than plasma fatty acids [52], whereas the FFQ assesses usual intake over the past 12 months. The difference in timeframe could result in some seasonal variation in dietary intake that is not reflected in the shorter-term measurement of erythrocyte fatty acids.

The results of this study illustrate a dose-dependent bias in the FFQ fatty acid data and the corresponding erythrocyte data. The Bland–Altman plots for ALA and LA suggest a ‘biphasic’ relationship between the biases of the two measures, which means one measure overestimates the other when the magnitude of the measure is large but conversely underestimates the other when the magnitude of the measure is small [53]. Based on the linear regression equation in Bland–Altman plots, we found that the intercept between EPA and DHA is much closer to zero. This means that the bias between the two measurements is monophasic, that is, it only occurs when the magnitude of the measurement is large. This suggests the FFQ tool is likely to have less measurement bias in identifying consumers with low intakes than those with high intakes, similar to the conclusions obtained using quartile cross-classification analysis. A study of FFQ and RBC data from Switzerland [46] found that the FFQ placed participants in the same or adjacent quartiles with an accuracy of between 70 and 87% for different fatty acids, with DHA having the highest accuracy, followed by EPA. In our results, cross-classification analysis resulted in the best performance for DHA, although Kappa statistics showed poor agreement.

Our study has some strengths, including a large amount of dietary and biomarker data, making this one of the largest studies of this type in adolescents. In Australia, most studies that investigated FFQ and erythrocyte fatty acids had sample sizes of less than 60 [10, 47]. We used a specifically designed and validated FFQ for the parent to complete in this age group, allowing us to better record usual dietary intake that is particularly beneficial when collecting information on foods such as fish and seafood as we have a complete fish database that records the amount of EPA and DHA in fish consumed [15].

Our study was limited by the time frame of the assessments, with the 12 month time period represented by the FFQ longer than the 3 month time period represented by the erythrocyte data. There is also a limitation that our study did not capture information on the consumption of dietary fatty acid supplements and therefore this study may not be representative of the population if consuming dietary fatty acid supplements.

Potential areas for future research include examining whether external factors such as other dietary factors, along with other modifiable lifestyle factors such as alcohol and smoking, influence the strength of the associations between fatty acid intake and erythrocyte biomarkers. In addition, this study tested the valid parameters of the FFQ data against the erythrocyte data; however, reproducibility was not tested. It would also be interesting to investigate whether this association persists or changes with age.

## Conclusions

In conclusion, poor to fair correlations were found between the FFQ and erythrocyte data for DPA, DHA and LA. The Bland–Altman plots suggest poor agreement and a dose-dependent bias between the two measures.

## Supplementary information

Supplementary file

## Data Availability

The data that support the findings of this study are available from the Raine Study (https://rainestudy.org.au/) but restrictions apply to the availability of these data, which were used under license for the current study, and so are not publicly available. Data are however available from the authors upon reasonable request and with permission of the Raine Study (https://rainestudy.org.au/).
