# Supplementary material for: Relationship between dietary intake and erythrocyte PUFA in adolescents from a Western Australian cohort
Source: Eur J Clin Nutr. 2022 Oct 7;77(2):283–91. doi: 10.1038/s41430-022-01219-x (PMC9908540; doi:10.1038/s41430-022-01219-x)
Supplement: Supplementary file 1 — Supplementary file [file 41430_2022_1219_MOESM1_ESM.docx]

Supplementary table 1: Mean (SD) and IQR values for intake grams per day & percentage of total fat and erythrocyte fatty acid in males (n=603)

| Fatty acids (total and subtypes) | Male(n=603) | | | | Female(n=552) | | | |  |
| --- | --- | --- | --- | --- | --- | --- | --- | --- | --- |
|  | Mean | SD | Median | IQR | Mean | SD | Median | IQR | P-value Difference between gender |
| FFQ data |  |  |  |  |  |  |  |  |  |
| n-3 PUFA, g/d | 1.38 | 0.66 | 1.24 | 0.96-1.62 | 1.20 | 0.59 | 1.04 | 0.81-1.45 | 0.200 |
| ALA, g/d | 1.12 | 0.62 | 0.97 | 0.75-1.29 | 0.95 | 0.59 | 0.81 | 0.61-1.13 | 0.857 |
| EPA, mg/d | 75.72 | 38.6 | 68.0 | 48.7-96.0 | 65.7 | 35.8 | 60.5 | 41.2-84.1 | 0.159 |
| DPA mg/d | 108 | 53.6 | 98.3 | 70.5-140 | 92.3 | 50.6 | 86.4 | 56.8-116 | 0.479 |
| DHA, mg/d | 78.6 | 58.7 | 66.2 | 40.5-101 | 73.2 | 56.1 | 59.5 | 33.82-96.1 | 0.062 |
| n-6 PUFA, g/d | 12.5 | 6.31 | 11.2 | 7.77-16.2 | 10.9 | 5.80 | 9.38 | 6.79-14.16 | 0.141 |
| LA g/d | 12.2 | 6.28 | 10.9 | 7.43-15.9 | 10.7 | 5.76 | 9.17 | 6.51-13.9 | 0.134 |
| AA mg/d | 186 | 82.6 | 173 | 128-230 | 156 | 77.6 | 145 | 102-191 | 0.999 |
| Erythrocyte data, % |  |  |  |  |  |  |  |  |  |
| n-3 PUFA | 8.47 | 1.24 | 8.55 | 7.78-9.22 | 8.58 | 1.19 | 8.59 | 7.93-9.22 | 0.165 |
| ALA | 0.61 | 0.22 | 0.58 | 0.52-0.64 | 0.63 | 0.19 | 0.61 | 0.54-0.67 | <0.001 |
| EPA | 0.69 | 0.18 | 0.69 | 0.57-0.79 | 0.70 | 0.19 | 0.70 | 0.59-0.81 | 0.181 |
| DPA | 2.38 | 0.40 | 2.38 | 2.16-2.61 | 2.40 | 0.39 | 2.39 | 2.17-2.63 | 0.528 |
| DHA | 4.19 | 0.98 | 4.18 | 3.61-4.77 | 4.22 | 0.92 | 4.21 | 3.67-4.76 | 0.451 |
| n-6 PUFA | 33.0 | 2.57 | 33.6 | 32.0-34.5 | 33.3 | 2.57 | 33.9 | 32.7-34.7 | 0.006 |
| LA | 9.94 | 1.05 | 9.92 | 9.29-10.6 | 9.97 | 1.00 | 9.91 | 9.36-10.6 | 0.480 |
| AA | 13.2 | 1.94 | 13.6 | 12.5-14.4 | 13.5 | 1.97 | 13.9 | 12.9-14.6 | 0.002 |

Footnotes:

AA, arachidonic acid; ALA, α-linolenic acid; DHA, docosahexaenoic acid; DPA, docosapentaenoic acid; EPA, eicosapentaenoic acid; IQR, Interquartile range; LA, linoleic acid; n-3 PUFA, Omega 3 polyunsaturated fatty acids; n-6 PUFA, Omega 6 polyunsaturated fatty acids; SD, standard deviation. P value tested by Wilcoxon Man-Whitney test, FFQ data use energy adjusted intake.

Supplementary table 2: Daily intake and percentage contribution of n-6, n-3 PUFA and LC PUFA intakes by food group as estimated from FFQ in males (n =603)

| Food groups | n-3 PUFA | | | | n-6 PUFA | | | | n-3 LC PUFA | | | |
| --- | --- | --- | --- | --- | --- | --- | --- | --- | --- | --- | --- | --- |
|  | g/d | | % | | g/d | | % | | mg/d | | % | |
|  | Median | IQR | Mean | sd | Median | IQR | Mean | sd | Median | IQR | Mean | sd |
| cereals, breads | 0.07 | 0.05-0.10 | 6.55 | 3.48 | 1.23 | 0.89-1.65 | 12.34 | 7.16 | 0.00 | 0.00-0.00 | 0.15 | 1.68 |
| cakes, biscuits, honey, jam | 0.02 | 0.01-0.04 | 2.37 | 2.42 | 0.19 | 0.09-0.36 | 2.40 | 2.72 | 0.13 | 0.00-0.29 | 0.16 | 2.04 |
| desserts, ice cream | 0.02 | 0.01-0.04 | 2.58 | 2.91 | 0.05 | 0.02-0.10 | 0.78 | 1.08 | 0.00 | 0.00-0.00 | 0.00 | 0.03 |
| dairy, soy products | 0.14 | 0.07-0.23 | 12.72 | 8.74 | 0.26 | 0.13-0.45 | 3.22 | 3.71 | 0.00 | 0.00-0.00 | 0.00 | 0.00 |
| beverages, sugar, milo | 0.00 | 0.00-0.00 | 0.05 | 0.35 | 0.00 | 0.00-0.01 | 0.10 | 0.34 | 0.00 | 0.00-0.00 | 0.00 | 0.00 |
| eggs | 0.01 | 0.01-0.03 | 1.37 | 1.32 | 0.19 | 0.07-0.37 | 2.39 | 2.50 | 5.43 | 2.11-10.3 | 1.60 | 1.83 |
| red meat, pork | 0.13 | 0.07-0.20 | 11.46 | 7.70 | 0.31 | 0.18-0.53 | 3.71 | 3.39 | 69.0 | 37.9-110 | 15.93 | 8.89 |
| sausages, ham, bacon | 0.05 | 0.04-0.09 | 5.34 | 3.89 | 0.44 | 0.27-0.75 | 5.23 | 4.38 | 9.64 | 5.46-15.4 | 2.70 | 2.43 |
| takeaway foods (not hot chips) | 0.09 | 0.05-0.13 | 8.17 | 5.74 | 0.40 | 0.25-0.61 | 4.52 | 3.73 | 12.3 | 6.19-19.0 | 3.44 | 3.40 |
| savoury dishes, soup, stews | 0.15 | 0.08-0.23 | 13.78 | 9.78 | 0.56 | 0.33-0.85 | 6.29 | 5.30 | 66.8 | 33.5-112 | 16.18 | 9.28 |
| chicken, fish (excl fresh fish) | 0.05 | 0.03-0.10 | 5.46 | 5.33 | 0.45 | 0.23-0.80 | 5.55 | 5.50 | 22.9 | 10.3-42.2 | 6.39 | 5.71 |
| canned, pickled veg, coleslaw | 0.00 | 0.00-0.01 | 0.38 | 0.72 | 0.10 | 0.02-0.28 | 1.66 | 2.14 | 0.00 | 0.00-0.00 | 0.00 | 0.00 |
| cooked vegetables, hot chips | 0.04 | 0.02-0.06 | 3.43 | 2.28 | 0.51 | 0.31-0.73 | 5.19 | 3.40 | 0.00 | 0.00-0.00 | 0.00 | 0.00 |
| salad vegetables, veg juice | 0.00 | 0.00-0.00 | 0.00 | 0.00 | 0.00 | 0.00-0.00 | 0.28 | 0.90 | 0.00 | 0.00-0.00 | 0.00 | 0.00 |
| fruit and fruit juice | 0.00 | 0.00-0.00 | 0.00 | 0.00 | 0.00 | 0.00-0.00 | 0.00 | 0.00 | 0.00 | 0.00-0.00 | 0.00 | 0.00 |
| crisps, snack foods, nuts, seeds | 0.00 | 0.00-0.04 | 4.12 | 10.89 | 0.43 | 0.14-1.01 | 7.37 | 10.52 | 0.00 | 0.00-0.00 | 0.00 | 0.00 |
| confectionery, ice cream bars, icy pole | 0.02 | 0.01-0.03 | 1.96 | 1.66 | 0.38 | 0.17-0.65 | 4.58 | 4.78 | 0.00 | 0.00-0.00 | 0.00 | 0.00 |
| sauce, dressing, dips, spreads | 0.00 | 0.00-0.01 | 0.51 | 1.03 | 0.33 | 0.00-0.75 | 4.84 | 6.64 | 0.00 | 0.00-0.00 | 0.00 | 0.00 |
| butter, margarine | 0.19 | 0.09-0.33 | 18.33 | 15.15 | 3.55 | 0.31-7.69 | 29.56 | 23.34 | 0.00 | 0.00-0.00 | 0.00 | 0.00 |
| fresh fish | 0.01 | 0.00-0.03 | 1.40 | 1.68 | 0.00 | 0.00-0.00 | 0.02 | 0.04 | 240 | 175-327 | 53.44 | 3.92 |

Footnotes:

IQR, Interquartile range; n-3 PUFA, Omega 3 polyunsaturated fatty acids; n-6 PUFA, Omega 6 polyunsaturated fatty acids; SD, standard deviation. LC PUFA, long chain omega 3 polyunsaturated fatty acids.Supplementary table 3: Daily intake and percentage contribution of n-6, n-3 PUFA and LC PUFA intakes by food group as estimated from FFQ in females (n =552)

| Food groups | n-3 PUFA | | | | n-6 PUFA | | | | n-3 LC PUFA | | | |
| --- | --- | --- | --- | --- | --- | --- | --- | --- | --- | --- | --- | --- |
|  | g/d | | % | | g/d | | % | | mg/d | | % | |
|  | Median | IQR | Mean | sd | Median | IQR | Mean | sd | Median | IQR | Mean | sd |
| cereals, breads | 0.06 | 0.04-0.08 | 6.41 | 4.08 | 1.02 | 0.71-1.38 | 11.69 | 6.90 | 0.00 | 0.00-0.00 | 0.16 | 1.54 |
| cakes, biscuits, honey, jam | 0.02 | 0.01-0.04 | 2.14 | 2.08 | 0.17 | 0.08-0.32 | 2.11 | 2.05 | 0.13 | 0.00-0.29 | 0.17 | 2.13 |
| desserts, ice cream | 0.02 | 0.01-0.03 | 2.14 | 2.16 | 0.05 | 0.02-0.08 | 0.68 | 0.86 | 0.00 | 0.00-0.00 | 0.00 | 0.02 |
| dairy, soy products | 0.11 | 0.06-0.17 | 11.16 | 7.48 | 0.19 | 0.10-0.32 | 2.58 | 2.50 | 0.00 | 0.00-0.00 | 0.00 | 0.00 |
| beverages, sugar, milo | 0.00 | 0.00-0.00 | 0.01 | 0.11 | 0.00 | 0.00-0.01 | 0.06 | 0.10 | 0.00 | 0.00-0.00 | 0.00 | 0.00 |
| eggs | 0.01 | 0.00-0.02 | 1.30 | 1.23 | 0.16 | 0.06-0.30 | 2.13 | 2.08 | 4.38 | 1.96-8.41 | 1.84 | 4.29 |
| red meat, pork | 0.10 | 0.05-0.16 | 10.73 | 8.48 | 0.25 | 0.11-0.42 | 3.34 | 3.29 | 52.6 | 26.2-90.1 | 14.54 | 9.61 |
| sausages, ham, bacon | 0.04 | 0.02-0.07 | 4.74 | 3.56 | 0.34 | 0.18-0.61 | 4.69 | 4.09 | 7.11 | 3.78-11.3 | 2.39 | 2.88 |
| takeaway foods (not hot chips) | 0.06 | 0.03-0.11 | 7.26 | 5.54 | 0.28 | 0.15-0.49 | 3.90 | 3.45 | 7.92 | 3.96-17.0 | 2.93 | 3.01 |
| savoury dishes, soup, stews | 0.13 | 0.08-0.23 | 15.69 | 12.13 | 0.51 | 0.30-0.88 | 7.21 | 6.37 | 59.3 | 30.4-101 | 16.84 | 9.72 |
| chicken, fish (excl fresh fish | 0.06 | 0.02-0.11 | 6.81 | 6.97 | 0.47 | 0.17-0.89 | 6.74 | 7.00 | 23.0 | 8.63-44.7 | 7.66 | 7.04 |
| canned, pickled veg, coleslaw | 0.00 | 0.00-0.01 | 0.54 | 1.34 | 0.11 | 0.03-0.25 | 1.95 | 2.57 | 0.00 | 0.00-0.00 | 0.00 | 0.00 |
| cooked vegetables, hot chips | 0.03 | 0.02-0.06 | 3.82 | 2.67 | 0.45 | 0.296-0.69 | 5.46 | 3.60 | 0.00 | 0.00-0.00 | 0.00 | 0.00 |
| salad vegetables, veg juice | 0.00 | 0.00-0.00 | 0.00 | 0.05 | 0.00 | 0.00-0.00 | 0.75 | 1.89 | 0.00 | 0.00-0.01 | 0.00 | 0.00 |
| fruit and fruit juice | 0.00 | 0.00-0.00 | 0.00 | 0.00 | 0.00 | 0.00-0.00 | 0.00 | 0.00 | 0.00 | 0.00-0.00 | 0.00 | 0.00 |
| crisps, snack foods, nuts, seeds | 0.00 | 0.00-0.04 | 4.87 | 12.31 | 0.43 | 0.14-1.01 | 8.25 | 11.88 | 0.00 | 0.00-0.00 | 0.00 | 0.00 |
| confectionery, ice cream bars, icy pole | 0.02 | 0.01-0.03 | 2.28 | 1.92 | 0.37 | 0.15-0.64 | 5.02 | 5.08 | 0.00 | 0.00-0.00 | 0.00 | 0.00 |
| sauce, dressing, dips, spreads | 0.00 | 0.00-0.01 | 0.64 | 1.38 | 0.35 | 0.00-0.70 | 5.31 | 7.37 | 0.00 | 0.00-0.00 | 0.00 | 0.00 |
| butter, margarine | 0.17 | 0.07-0.28 | 17.98 | 15.64 | 2.69 | 0.23-6.02 | 28.10 | 22.85 | 0.00 | 0.00-0.00 | 0.00 | 0.00 |
| fresh fish | 0.01 | 0.00-0.02 | 1.45 | 2.01 | 0.00 | 0.00-0.00 | 0.02 | 0.05 | 213 | 146-295 | 53.46 | 4.65 |

Footnotes:

IQR, Interquartile range; n-3 PUFA, Omega 3 polyunsaturated fatty acids; n-6 PUFA, Omega 6 polyunsaturated fatty acids; SD, standard deviation. LC PUFA, long chain omega 3 polyunsaturated fatty acids.

Supplementary table 4: Correlation data at 14 year of age in the Raine Study: nutrient intake (g/day) against erythrocyte measures and percentage fatty acid intake from total fat and percentage of erythrocyte measures using an energy-adjusted rho (ρ) correlation in females (n=552).

| FFQ | Erythrocyte | | | | | | | | | | | |
| --- | --- | --- | --- | --- | --- | --- | --- | --- | --- | --- | --- | --- |
|  | n-6 PUFA | | | | n-3 PUFA | | | | | | | |
|  | LA | P | AA | P | ALA | P | EPA | P | DPA | P | DHA | P |
| n-3 PUFA | 0.051 | 0.232 | -0.087 | 0.041 | 0.020 | 0.642 | 0.071 | 0.096 | -0.041 | 0.339 | 0.141 | 0.001 |
| ALA | 0.042 | 0.322 | -0.082 | 0.055 | 0.042 | 0.329 | 0.065 | 0.127 | -0.043 | 0.314 | 0.078 | 0.068 |
| EPA | -0.038 | 0.368 | -0.046 | 0.283 | -0.017 | 0.694 | 0.018 | 0.682 | -0.070 | 0.100 | 0.142 | 0.001 |
| DPA | -0.015 | 0.727 | -0.043 | 0.314 | 0.009 | 0.834 | -0.058 | 0.174 | -0.093 | 0.029 | 0.036 | 0.405 |
| DHA | -0.009 | 0.829 | -0.054 | 0.207 | -0.030 | 0.483 | 0.170 | 0.001 | -0.045 | 0.296 | 0.341 | <0.001 |
| n-6 PUFA | 0.062 | 0.149 | -0.026 | 0.540 | -0.061 | 0.149 | -0.035 | 0.409 | -0.046 | 0.280 | 0.078 | 0.068 |
| LA | 0.063 | 0.139 | -0.026 | 0.543 | -0.062 | 0.145 | -0.031 | 0.463 | -0.043 | 0.309 | 0.080 | 0.068 |
| AA | -0.013 | 0.753 | -0.064 | 0.135 | 0.032 | 0.458 | -0.091 | 0.033 | -0.127 | 0.003 | 0.020 | 0.639 |

Footnote:

AA, arachidonic acid; ALA, α-linolenic acid; DHA, docosahexaenoic acid; DPA, docosapentaenoic acid; EPA, eicosapentaenoic acid; LA, linoleic acid; n-3 PUFA, Omega 3 polyunsaturated fatty acids; n-6 PUFA, Omega 6 polyunsaturated fatty acids;

Supplementary table 5: Correlation data at 14 year of age in the Raine Study: nutrient intake (g/day) against erythrocyte measures and percentage fatty acid intake from total fat and percentage of erythrocyte measures using an energy-adjusted rho (ρ) correlation in males (n=603).

| FFQ | Erythrocyte | | | | | | | | | | | |
| --- | --- | --- | --- | --- | --- | --- | --- | --- | --- | --- | --- | --- |
|  | n-6 PUFA | | | | n-3 PUFA | | | | | | | |
|  | LA | P | AA | P | ALA | P | EPA | P | DPA | P | DHA | P |
| n-3 PUFA | 0.096 | 0.019 | 0.006 | 0.881 | -0.089 | 0.028 | 0.085 | 0.058 | -0.018 | 0.662 | 0.090 | 0.027 |
| ALA | 0.111 | 0.006 | -0.020 | 0.621 | -0.064 | 0.117 | 0.085 | 0.036 | -0.030 | 0.469 | 0.017 | 0.679 |
| EPA | -0.031 | 0.450 | -0.039 | 0.342 | -0.048 | 0.238 | 0.038 | 0.353 | -0.059 | 0.148 | 0.192 | <0.001 |
| DPA | -0.024 | 0.561 | 0.020 | 0.623 | -0.062 | 0.131 | -0.003 | 0.939 | -0.055 | 0.177 | 0.125 | 0.002 |
| DHA | -0.042 | 0.307 | -0.068 | 0.095 | -0.070 | 0.087 | 0.118 | 0.004 | -0.113 | 0.006 | 0.290 | <0.001 |
| n-6 PUFA | 0.126 | 0.002 | -0.044 | 0.280 | -0.104 | 0.011 | -0.046 | 0.257 | -0.032 | 0.438 | -0.077 | 0.058 |
| LA | 0.125 | 0.002 | -0.044 | 0.279 | -0.101 | 0.013 | -0.046 | 0.257 | -0.032 | 0.438 | -0.077 | 0.058 |
| AA | -0.067 | 0.103 | 0.067 | 0.098 | -0.079 | 0.053 | -0.029 | 0.479 | -0.090 | 0.028 | 0.127 | 0.002 |

Footnote:

AA, arachidonic acid; ALA, α-linolenic acid; DHA, docosahexaenoic acid; DPA, docosapentaenoic acid; EPA, eicosapentaenoic acid; LA, linoleic acid; n-3 PUFA, Omega 3 polyunsaturated fatty acids; n-6 PUFA, Omega 6 polyunsaturated fatty acids;

Supplementary table 6. Dietary PUFA intakes classified into quintiles compared with quintiles of erythrocyte PUFA proportions with corresponding Cohen’s κ coefficients in males (n=603)

| FFQ and Erythrocyte PUFA | Same quintile (%) | Same or adjacent quintile (%) | Opposite quintile (%) | Cohen’s Kappa (κ) | | |
| --- | --- | --- | --- | --- | --- | --- |
|  |  |  |  | Cohen’s κ | 95% CI | P-value |
| n-3 PUFA | 23.05 | 53.90 | 7.46 | 0.040 | -0.017-0.097 | 0.167 |
| ALA | 17.58 | 51.74 | 8.46 | -0.034 | -0.087-0.026 | 0.206 |
| EPA | 22.22 | 56.05 | 7.96 | 0.034 | -0.023-0.090 | 0.241 |
| DPA | 18.08 | 50.75 | 6.80 | -0.043 | -0.097-0.012 | 0.122 |
| DHA | 27.53 | 61.03 | 3.48 | 0.180 | 0.123-0.236 | <0.001 |
| n-6 PUFA | 20.23 | 53.57 | 7.63 | 0.034 | -0.022-0.089 | 0.236 |
| LA | 22.22 | 55.39 | 7.63 | 0.073 | 0.017-0.129 | 0.010 |
| AA | 23.71 | 53.40 | 7.63 | 0.050 | -0.007-0.106 | 0.084 |

Footnote:

AA, arachidonic acid; ALA, α-linolenic acid; DHA, docosahexaenoic acid; DPA, docosapentaenoic acid; EPA, eicosapentaenoic acid; LA, linoleic acid; n-3 PUFA, Omega 3 polyunsaturated fatty acids; n-6 PUFA, Omega 6 polyunsaturated fatty acids;

Cohen's Kappa analysis using the weighted Kappa statistic (κ)

Supplementary table 7. Dietary PUFA intakes classified into quintiles compared with quintiles of erythrocyte PUFA proportions with corresponding Cohen’s κ coefficients in females (n=552)

| FFQ and Erythrocyte PUFA | Same quintile (%) | Same or adjacent quintile (%) | Opposite quintile (%) | Cohen’s Kappa (κ) | | |
| --- | --- | --- | --- | --- | --- | --- |
|  |  |  |  | Cohen’s κ | 95% CI | P-value |
| n-3 PUFA | 24.64 | 56.34 | 7.25 | 0.083 | 0.024-0.142 | 0.006 |
| ALA | 23.37 | 56.34 | 8.70 | 0.047 | -0.012-0.105 | 0.119 |
| EPA | 22.46 | 49.82 | 6.16 | 0.006 | -0.053-0.065 | 0.840 |
| DPA | 16.67 | 49.09 | 8.89 | -0.067 | -0.123—0.011 | 0.020 |
| DHA | 28.44 | 64.13 | 3.44 | 0.218 | 0.160-0.276 | <0.001 |
| n-6 PUFA | 22.28 | 51.81 | 8.70 | 0.000 | -0.058-0.058 | 0.991 |
| LA | 21.92 | 54.89 | 5.80 | 0.049 | -0.010-0.107 | 0.103 |
| AA | 18.84 | 47.64 | 9.24 | -0.050 | -0.106-0.007 | 0.086 |

Footnote:

AA, arachidonic acid; ALA, α-linolenic acid; DHA, docosahexaenoic acid; DPA, docosapentaenoic acid; EPA, eicosapentaenoic acid; LA, linoleic acid; n-3 PUFA, Omega 3 polyunsaturated fatty acids; n-6 PUFA, Omega 6 polyunsaturated fatty acids;

Cohen's Kappa analysis using the weighted Kappa statistic (κ)

**Supplementary figure**. Bland-Altman plot, showing 95% limits of agreement for total N-3,ALA, EPA, DPA, DHA, total N-6, LA, AA in male and female.

Supplementary figure legends: AA, arachidonic acid; ALA, α-linolenic acid; DHA, docosahexaenoic acid; DPA, docosapentaenoic acid; EPA, eicosapentaenoic acid; LA, linoleic acid; total N-3, Omega 3 polyunsaturated fatty acids; total N-6, Omega 6 polyunsaturated fatty acids, FFQ: Food Frequency Questionnaire
